# Nationwide trends in pulmonary rehabilitation and clinical testing practices for chronic obstructive pulmonary disease in Korea: a National Health Insurance Service database study

**DOI:** 10.3389/fresc.2026.1778935

**Published:** 2026-10-27

**Authors:** Sang-Hun Kim, Cho Hui Hong, Jinmi Kim, Saerom Kim, Soo Han Kim, Mi-Hyun Kim, Myung-Jun Shin, Tae Sung Park, Jong-Hwa Jeong, Yong Beom Shin

**Affiliations:** 1 Department of Rehabilitation Medicine, Biomedical Research Institute, Pusan National University Hospital, Pusan National University School of Medicine, Busan, Republic of Korea; 2 Biomedical Research Institute, Pusan National University Hospital, Busan, Republic of Korea; 3 Department of Biostatistics, Clinical Trial Center, Biomedical Research Institute, Pusan National University Hospital, Busan, Republic of Korea; 4 Department of Internal Medicine, Pusan National University Hospital, Pusan National University School of Medicine, Busan, Republic of Korea; 5 Department of Convergence Medical Institute of Technology, Pusan National University Hospital, Busan, Republic of Korea; 6 Department of Physical Therapy, Department of Rehabilitation Medicine, Pusan National University Hospital, Busan, Republic of Korea

**Keywords:** administrative claims data, cardiopulmonary exercise testing, chronic obstructive pulmonary disease, National Health Insurance Service, pulmonary rehabilitation

## Abstract

Pulmonary rehabilitation (PR) is an evidence-based intervention for chronic obstructive pulmonary disease (COPD) and has been covered by Korea's National Health Insurance Service since 2016; however, national trends in PR use and related clinical testing have not been fully characterized. We conducted a nationwide retrospective cohort study using NHIS claims data from 2017 to 2021 among adults aged ≥40 years with COPD. Claim-defined comprehensive PR utilization was defined as at least one MM440 claim, while MM290 claims were analyzed separately. We performed 1:1 propensity score matching by age, sex, and region to compare PR recipients with non-recipients. The annual implementation rate of comprehensive PR (MM440) increased from 3.9 to 11.6 per 100,000 population, and the proportion of patients receiving PR rose from 0.49% in 2017 to 1.56% in 2021, while the mean number of PR claims per patient declined. In 2021, 94.8% of MM440 recipients had fewer than eight recorded MM440 claims. After matching, PR recipients had higher hospitalization rates, were more frequently treated in tertiary hospitals, and had higher malignancy-related claim counts and greater eligibility for co-payment reduction than non-recipients. The annual ratios of maximal inspiratory/expiratory pressure testing and cardiopulmonary exercise testing claims to MM440 claims were 11.2% and 7.0%, respectively, in 2021. In conclusion, although claim-defined PR utilization for COPD in Korea increased, overall uptake remained low, and the use of objective functional assessments was infrequent. These findings highlight persistent gaps in access and objective quality measurement and provide evidence to inform future PR implementation strategies in Korea.

## Introduction

Chronic obstructive pulmonary disease (COPD) is a major cause of morbidity and mortality worldwide, including in Korea. Pulmonary rehabilitation (PR) is an evidence-based intervention proven to improve dyspnea, quality of life, and clinical outcomes in COPD (1–3). In recognition of its clinical value, the National Health Insurance Service (NHIS) began covering PR costs in 2016 to improve accessibility (4).

A recent nationwide cohort study using the Health Insurance Review and Assessment Service (HIRA) database reported that PR significantly reduced exacerbations and mortality among Korean patients with COPD, despite a participation rate of only 1.43% in 2019 (5). However, it remains unclear whether patients undergoing PR receive appropriate objective functional assessments, such as respiratory muscle strength testing, necessary for tailoring individualized programs (6, 7). To our knowledge, no study has investigated these practices in COPD using NHIS data. Compared with HIRA, the NHIS database offers nationwide coverage, includes patient-level socioeconomic information, and reflects more recent trends. While NHIS-based studies have examined PR use in other populations, such as patients with lung cancer (8), the extent of PR utilization and incorporation of functional assessments for COPD remain unexplored. Therefore, this study aimed to provide updated, nationally representative estimates of PR utilization and the application of key clinical testing practices among Korean patients with COPD, focusing on implementation gaps in access, continuity, and quality measurement that are relevant across health systems.

## Materials and methods

### Study design and data source

This retrospective, population-based cohort study utilized administrative claims data from the NHIS database covering the Korean population. A tailored dataset for January 1, 2017, to December 31, 2021, was obtained from the NHIS Data Sharing Service.

### Patient population and definitions

Eligible participants were adults aged ≥40 years with COPD, identified based on the Korean Classification of Diseases, 8th revision (KCD-8) diagnosis codes J43–J44, excluding J43.0 (unilateral emphysema and MacLeod's syndrome). Codes listed as either primary or secondary diagnoses were accepted. This diagnosis-code–based definition was used to identify a broad claims-defined COPD population. Medication information was not available in the tailored dataset, and spirometric confirmation, symptom burden, and disease severity could not be ascertained from the NHIS claims data. Individuals with missing age or sex data were excluded. The study population construction, eligibility criteria, and annual analytic populations are summarized in Supplementary Figure S1. For the 2021 propensity score–matched analysis, PR exposure was defined as at least one MM440 claim. MM290 was analyzed separately and was not included in this exposure definition.

### Definition of PR and related clinical testing

Two rehabilitation-related NHIS claim codes were examined separately: MM440 (a comprehensive, one-hour exercise-based rehabilitation session combining aerobic, strength, and flexibility training) and MM290 (therapeutic breathing exercises, including breathing retraining and airway clearance techniques). Related clinical testing was identified using the following claim codes: FX663 [maximal inspiratory/expiratory pressure (MIP/MEP) testing to assess respiratory muscle strength], EX780 (handgrip strength testing as a measure of sarcopenia and frailty), F6012 [cardiopulmonary exercise testing (CPET) with gas analysis], and E6543 (CPET without gas analysis) (9, 10). Because the NHIS claims data do not capture treatment frequency, total program duration, completion status, or whether claims belonged to a single structured PR program, PR claims were interpreted as claim-defined utilization rather than confirmed participation in a structured or completed PR program. The number of PR claims was used only as an indirect proxy for continuity because repeated reimbursed PR claims were the only available claim-based indicator of repeated PR encounters. For each calendar year, the number of claims for each clinical testing code was divided by the total number of MM440 claims in the same year. These values represent annual claim-count ratios and do not imply temporal linkage between a testing claim and a specific MM440 session. Because discrete PR episodes could not be identified in the claims data, the temporal relationship between PR claims and testing claims could not be reliably determined; therefore, testing claims could not be classified as occurring before, during, or after rehabilitation. For the descriptive analysis of medical specialty distribution, MM440 claims were classified by billing specialty, and annual proportions were calculated using the total number of MM440 claims included in this analysis for each calendar year.

### Statistical analysis

Categorical variables were expressed as numbers (%) and continuous variables as means ± standard deviations. Group comparisons used independent t-tests (continuous) or chi-square tests (categorical). All analyses were performed using complete-case data for the variables included in each analysis. We performed 1:1 propensity score matching (PSM) using a logistic regression model for age, sex, and region, applying nearest-neighbor matching without replacement. No caliper restriction was applied (caliper = NULL). The matching model was limited to these demographic and geographic variables because the purpose of the analysis was to provide a descriptive comparison of healthcare utilization and clinical characteristics after accounting for basic demographic and geographic differences, rather than to estimate causal effects or identify independent determinants of PR use. Standardized mean difference (SMD) < 0.1 indicated adequate balance for the matched variables, and post-matching balance was assessed using SMDs (11). Analyses were conducted using SAS Enterprise Guide (version 7.15) and R (version 4.0.3, ‘MatchIt’ package). A two-tailed *P* value <0.05 was considered statistically significant.

### Ethics statement

The study was approved by the Institutional Review Board of Pusan National University Hospital (IRB No. 2210-001-119). The requirement for informed consent was waived because only anonymized secondary data were used.

## Results

### Baseline characteristics of the study population

In the propensity score–matched cohort of 11,894 patients with COPD (5,947 patients with ≥1 MM440 claim and 5,947 matched non-recipients), sex and age were similar between the groups after matching (Table 1), whereas several non-matched variables remained imbalanced. Insurance characteristics remained imbalanced after matching. The PR group had a higher proportion of patients enrolled in the Co-payment Reduction Program (48.7% vs. 35.1%; SMD 0.279), whereas the non-PR group included more Medical Aid beneficiaries (23.6% vs. 9.7%; SMD 0.380). The PR group was more likely to receive care at tertiary hospitals (81.2% vs. 50.0%; SMD 0.695) and had higher healthcare utilization, including more admissions (1.58 vs. 0.88; SMD 0.301) and longer cumulative hospital stays for respiratory diseases (e.g., 49.25 vs. 28.29 days; SMD 0.378). Among comorbidities, malignancy showed a notable between-group difference, with higher malignancy-related claim counts in the PR group (11.9 vs. 6.2 claims per patient per year; SMD 0.327), whereas most other comorbidities had SMDs <0.1.

**Table 1:** Baseline characteristics of patients with COPD in 2021, stratified by PR and non-PR groups.

Variables	Overall (*n* = 380,320)	PR group (*n* = 5,947)	Non-PR group (*n* = 374,373)	SMD	PR group (matched *n* = 5,947)	Non-PR group (matched *n* = 5,947)	SMD
Sex, male	268,560 (70.6)	4,488 (75.5)	264,072 (70.5)	0.111	4,488 (75.5)	4,490 (75.5)	0.001
Mean age, yr	72.66 ± 10.58	71.20 ± 9.38	72.69 ± 10.59	0.149	71.20 ± 9.38	71.31 ± 9.56	0.012
Age, yr				0.209			0.005
≤ 59	42,570 (11.2)	660 (11.1)	41,910 (11.2)		660 (11.1)	652 (11.0)	
60–69	96,206 (25.3)	1,798 (30.2)	94,408 (25.2)		1,798 (30.2)	1,796 (30.2)	
70–79	135,051 (35.5)	2,324 (39.1)	132,727 (35.5)		2,324 (39.1)	2,327 (39.1)	
≥ 80	106,493 (28.0)	1,165 (19.6)	105,328 (28.1)		1,165 (19.6)	1,172 (19.7)	
Insurance type							
National Health Insurance (NHI)—Self-employed	113,243 (29.8)	1,860 (31.3)	111,383 (29.8)	0.033	1,860 (31.3)	1,616 (27.2)	0.09
National Health Insurance (NHI)—Employee	219,874 (57.8)	3,511 (59.0)	216,363 (57.8)	0.025	3,511 (59.0)	2,928 (49.3)	0.198
Co-payment Reduction Program	59,961 (15.8)	2,896 (48.7)	57,065 (15.2)	0.768	2,896 (48.7)	2,085 (35.1)	0.279
Medical aid	47,135 (12.4)	575 (9.7)	46,560 (12.4)	0.088	575 (9.7)	1,401 (23.6)	0.38
Hospital type							
Tertiary hospital	131,962 (34.7)	4,828 (81.2)	127,134 (34.0)	1.088	4,828 (81.2)	2,972 (50.0)	0.695
General hospital	160,703 (42.3)	2,601 (43.7)	158,102 (42.2)	0.03	2,601 (43.7)	2,731 (45.9)	0.044
Secondary hospital	50,806 (13.4)	465 (7.8)	50,341 (13.4)	0.183	465 (7.8)	692 (11.6)	0.129
Clinic	92,595 (24.3)	551 (9.3)	92,044 (24.6)	0.417	551 (9.3)	1,125 (18.9)	0.28
Number of outpatient visits	4.67 ± 7.37	5.62 ± 9.94	4.65 ± 7.32	0.111	5.62 ± 9.94	4.83 ± 7.46	0.091
No. of admissions	0.78 ± 2.32	1.58 ± 2.51	0.76 ± 2.32	0.338	1.58 ± 2.51	0.88 ± 2.15	0.301
Length of hospital stay, days							
Pneumonia	5.88 ± 25.47	15.96 ± 39.93	5.72 ± 25.14	0.307	15.96 ± 39.93	6.03 ± 23.47	0.303
Chronic pulmonary disease	22.65 ± 52.65	38.07 ± 52.60	22.41 ± 52.62	0.298	38.07 ± 52.60	22.02 ± 44.13	0.331
All respiratory diseases	28.24 ± 55.67	49.25 ± 61.22	27.90 ± 55.52	0.365	49.25 ± 61.22	28.29 ± 48.89	0.378
Comorbidity							
Myocardial infarction	0.19 ± 1.83	0.29 ± 2.90	0.19 ± 1.81	0.043	0.29 ± 2.90	0.19 ± 1.26	0.045
Congestive heart failure	1.29 ± 4.95	1.59 ± 5.47	1.29 ± 4.94	0.057	1.59 ± 5.47	1.35 ± 5.09	0.045
Peripheral vascular disease	1.74 ± 6.27	1.43 ± 6.71	1.75 ± 6.27	0.049	1.43 ± 6.71	1.71 ± 5.86	0.044
Cerebrovascular disease	1.56 ± 5.57	1.55 ± 5.75	1.56 ± 5.56	0.002	1.55 ± 5.75	1.71 ± 6.47	0.027
Dementia	1.49 ± 4.99	0.82 ± 3.18	1.50 ± 5.01	0.161	0.82 ± 3.18	1.21 ± 5.55	0.085
Rheumatologic disease	0.33 ± 2.75	0.52 ± 3.73	0.33 ± 2.74	0.059	0.52 ± 3.73	0.47 ± 2.86	0.015
Peptic ulcer disease	1.55 ± 5.74	1.55 ± 3.67	1.55 ± 5.77	<0.001	1.55 ± 3.67	1.64 ± 5.81	0.019
Mild liver disease	1.40 ± 4.96	1.62 ± 4.85	1.39 ± 4.96	0.045	1.62 ± 4.85	1.79 ± 6.09	0.032
Diabetes without complication	3.22 ± 8.66	3.93 ± 9.83	3.20 ± 8.64	0.078	3.93 ± 9.83	3.78 ± 9.67	0.015
Diabetes with complication	1.33 ± 8.13	1.28 ± 8.06	1.33 ± 8.13	0.006	1.28 ± 8.06	1.51 ± 8.75	0.027
Hemiplegia or paraplegia	0.28 ± 3.11	0.38 ± 3.58	0.28 ± 3.10	0.031	0.38 ± 3.58	0.36 ± 4.28	0.004
Renal disease	1.52 ± 12.90	1.97 ± 14.61	1.51 ± 12.87	0.033	1.97 ± 14.61	2.27 ± 15.89	0.02
Any malignancy	3.22 ± 10.31	11.93 ± 20.73	3.08 ± 9.99	0.544	11.93 ± 20.73	6.16 ± 13.95	0.327
Moderate or severe liver disease	0.03 ± 0.68	0.06 ± 0.73	0.03 ± 0.68	0.033	0.06 ± 0.73	0.04 ± 0.63	0.017
Metastatic solid tumor	0.27 ± 2.29	0.69 ± 3.53	0.26 ± 2.26	0.144	0.69 ± 3.53	0.62 ± 3.82	0.02

Values are presented as numbers (%) or mean ± standard deviation. Values for comorbidities are presented as the mean number of claims per patient per year (mean ± SD) for each specific condition. Propensity score matching was performed on sex, age, and region. The PR group was defined as patients with at least one MM440 claim in 2021; MM290 claims were not included in this definition. PR, pulmonary rehabilitation; SMD, standardized mean difference.

### Trends in PR utilization

Figure 1 summarizes the annual number of patients receiving PR and the mean number of PR claims per patient. In Figure 1A, the number of patients receiving MM440 increased from 2,008 in 2017 to 5,947 in 2021, while the mean number of claims per patient decreased modestly from 3.0 to 2.6. In Figure 1B, the number of patients receiving MM290 increased to 2,087 in 2019 and then decreased to 1,572 in 2021, with the mean number of claims per patient remaining relatively stable (1.9–2.2). In 2021, most MM440 recipients had fewer than eight claims (5,638/5,947; 94.8%), whereas 145 (2.4%) had 8–14 claims and 164 (2.8%) had >14 claims. These categories describe recorded claim frequency and do not indicate adherence to or completion of a structured PR program. Figure 2 presents annual implementation rates expressed as the number of patients prescribed PR per 100,000 persons in the general population. In Figure 2A, the implementation rate for MM440 increased from 3.9 to 11.6 per 100,000 persons between 2017 and 2021, with a relative plateau between 2019 and 2020. In Figure 2B, the implementation rate for MM290 increased to 4.1 per 100,000 persons in 2019 and then declined to 3.1 per 100,000 persons in 2021, indicating an overall decrease after 2019.

**Figure 1:**
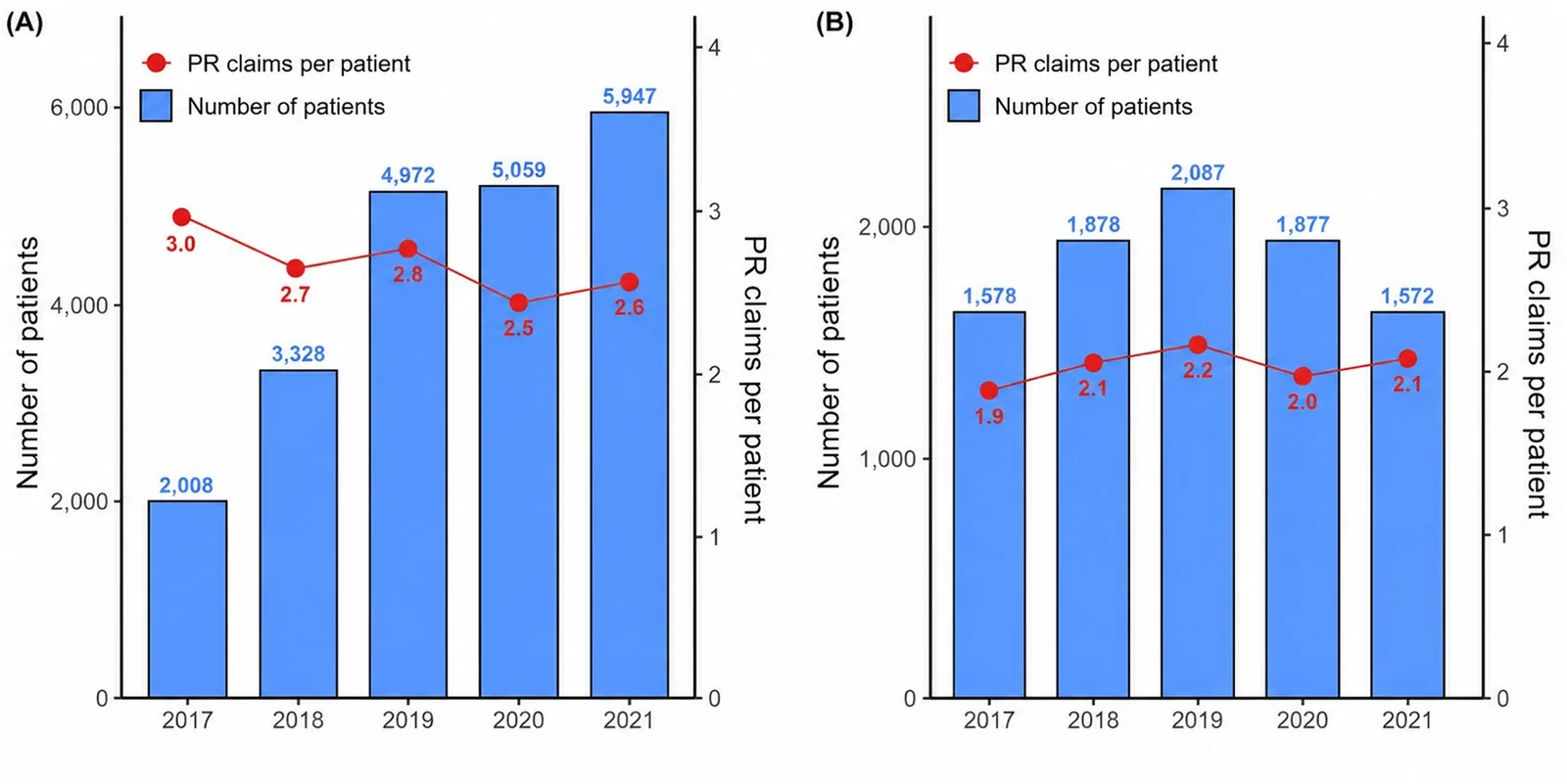
Yearly number of patients with COPD who received PR in Korea from 2017 to 2021 and the mean number of PR claims per patient (red line). **(A)** Number of patients prescribed MM440-based PR. **(B)** Number of patients prescribed MM290-based PR. PR, pulmonary rehabilitation; COPD, chronic obstructive pulmonary disease.

**Figure 2:**
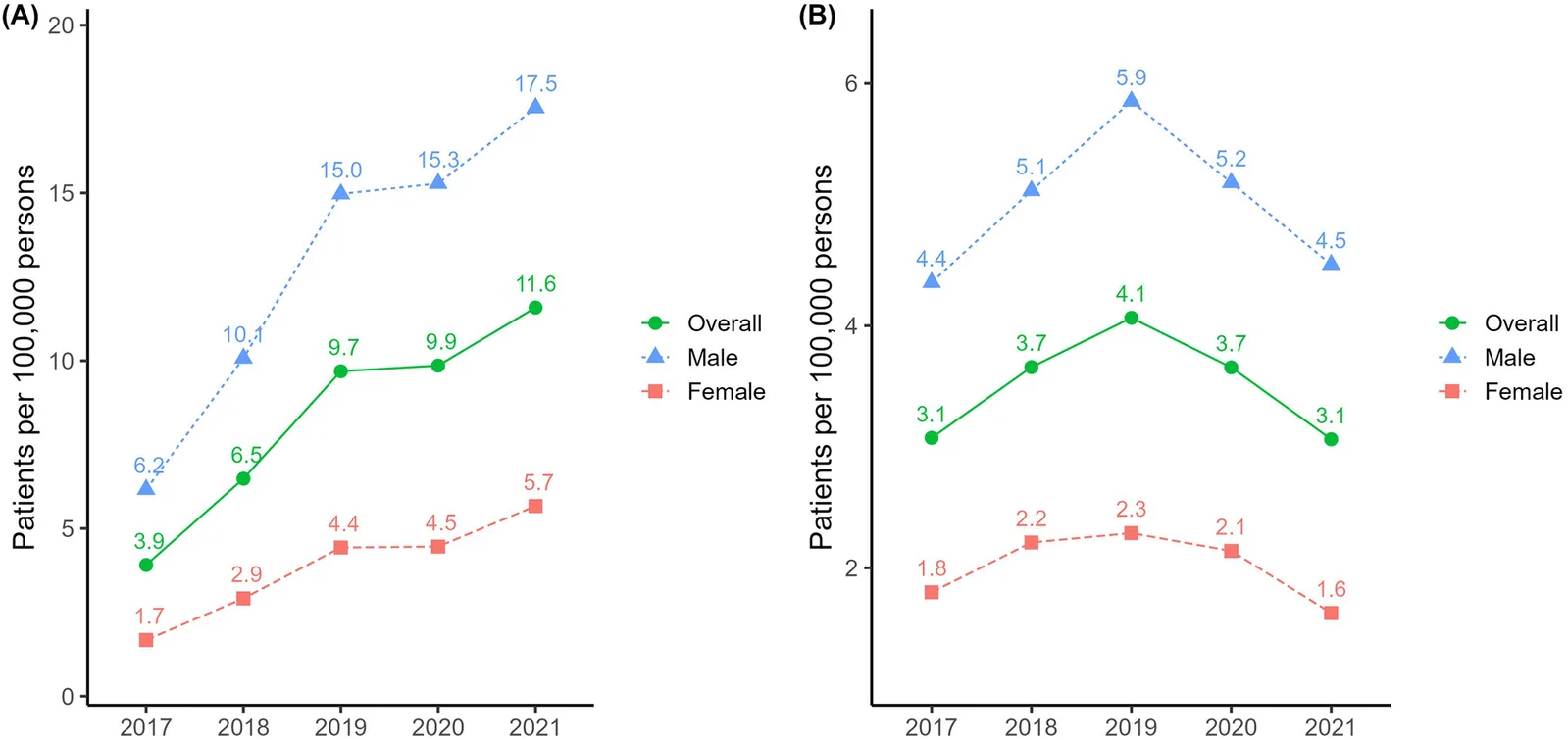
Annual trend of PR implementation rates among patients with COPD in Korea, expressed as the number of patients prescribed PR per 100,000 persons in the general population. **(A)** MM440 billing code. **(B)** MM290 billing code. PR, pulmonary rehabilitation; COPD, chronic obstructive pulmonary disease.

### Regional distribution of PR services

Regional PR implementation rates, defined as the number of patients prescribed MM440-based PR per 100,000 population, varied substantially across Korea (Figure 3). In 2017, the highest rates were observed in Jeonbuk, Busan, and Ulsan, followed by Seoul. By 2021, Gangwon recorded the highest implementation rate, followed by Seoul, Busan, Jeonbuk, and Daejeon; several other provinces also showed modest increases.

**Figure 3:**
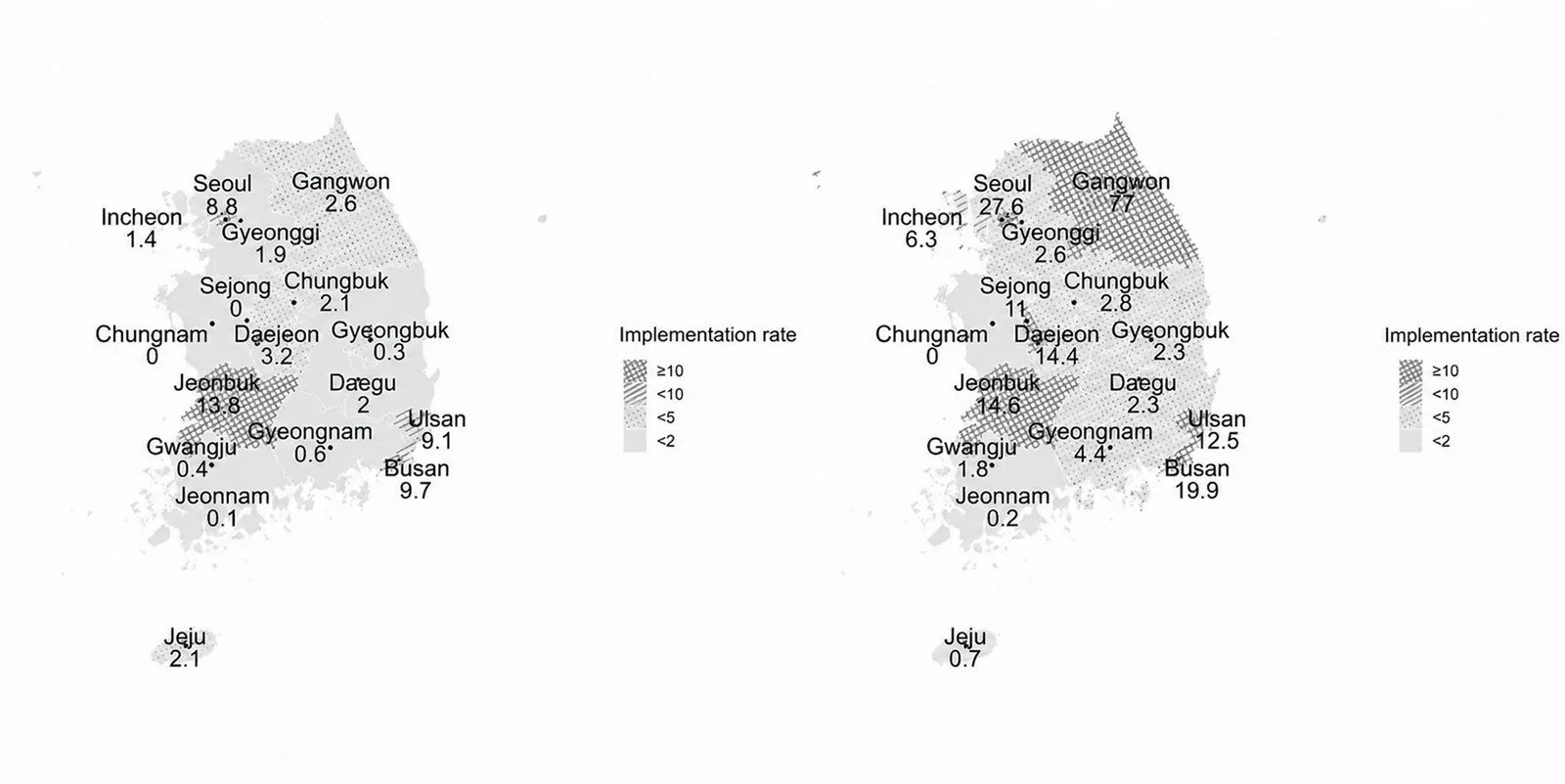
Regional distribution of PR implementation rates (MM440) in 2017 and 2021. PR, pulmonary rehabilitation. Implementation rate indicates the number of patients prescribed MM440-based PR per 100,000 persons.

### Utilization of related clinical testing

The annual numbers of related clinical testing claims and their ratios to MM440 claims are shown in Table 2. The number of FX663 claims rose from 923 in 2017 to 1,754 in 2021, and F6012 claims increased from 507 to 1,107 over the same period. EX780 claims also increased from 3,913 to 5,311. In 2021, the ratios of FX663 and F6012 claims to MM440 claims were 11.2% and 7.0%, respectively (Table 2). These values represent annual claim-count ratios rather than patient-level testing rates and do not imply temporal linkage to individual MM440 sessions.

**Table 2:** Annual counts and ratios of related clinical testing claims to MM440 claims among patients with COPD, 2017–2021.

Code	2017	2018	2019	2020	2021
PR, MM440	6,094	9,015	14,014	12,817	15,727
Maximal inspiratory/expiratory pressure, FX663	923 (15.1%)	1,224 (13.6%)	1,707 (12.2%)	1,515 (11.8%)	1,754 (11.2%)
Handgrip strength test, EX780	3,913 (64.2%)	4,249 (47.1%)	4,643 (33.1%)	5,085 (39.7%)	5,311 (33.8%)
CPET–with gas analysis, F6012	507 (8.3%)	637 (7.1%)	854 (6.1%)	1,001 (7.8%)	1,107 (7.0%)
CPET–without gas analysis, E6543	6,040 (99.1%)	6,271 (69.6%)	6,304 (45.0%)	5,407 (42.2%)	5,501 (35.0%)

Values are presented as number (%). Percentages were calculated as the number of claims for each assessment code divided by the total number of MM440 claims within the same calendar year. These ratios do not indicate temporal linkage to individual MM440 sessions and should not be interpreted as patient-level testing rates. PR, pulmonary rehabilitation; CPET, cardiopulmonary exercise testing.

### Distribution of PR claims by medical specialty

The proportional distribution of MM440 claims by medical specialty is shown in Figure 4. Rehabilitation Medicine accounted for the largest proportion throughout the study period, although its share decreased from 66.2% in 2017 to 54.7% in 2021. In contrast, the proportions attributed to Internal Medicine and Thoracic Surgery increased from 29.2% to 34.6% and from 3.3% to 6.1%, respectively.

**Figure 4:**
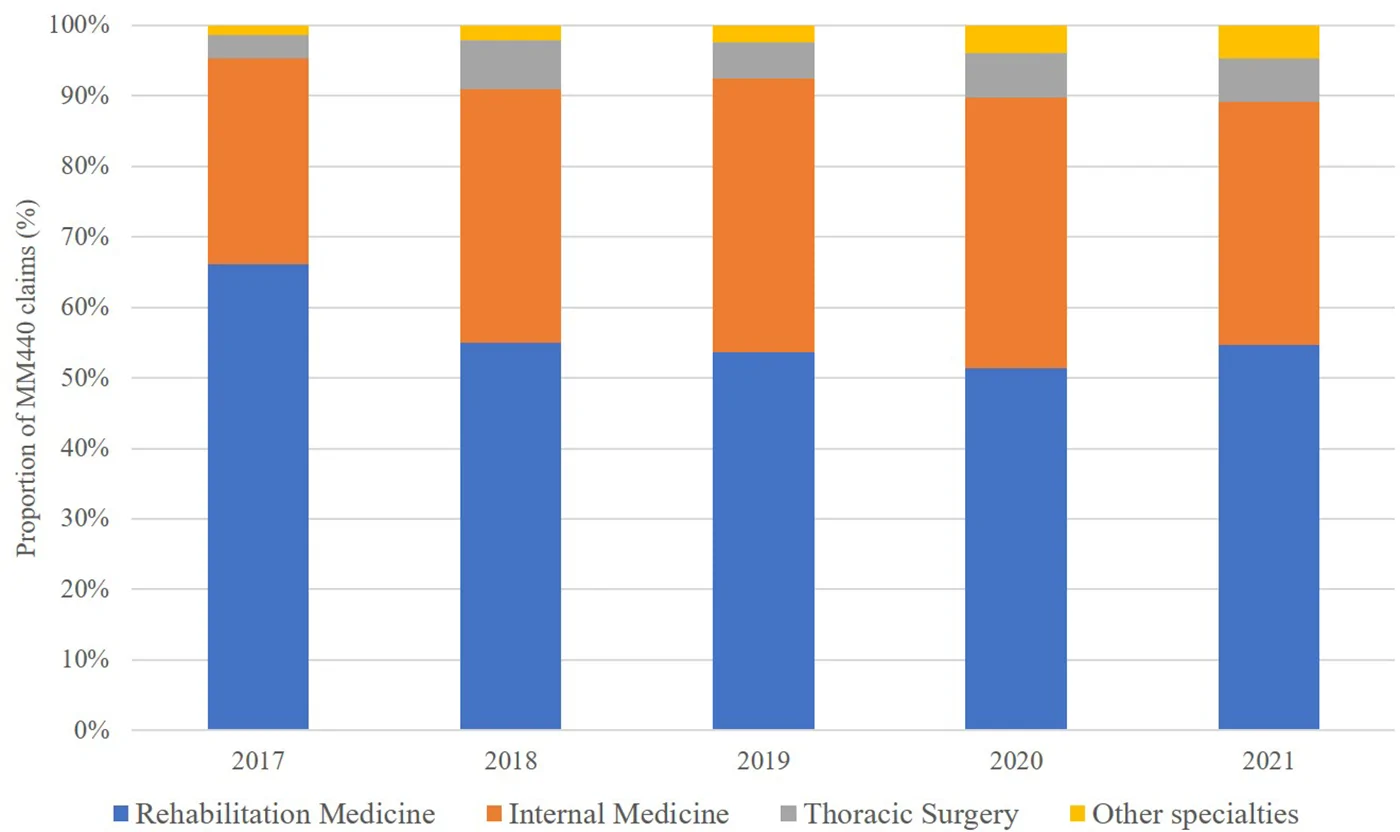
Annual proportional distribution of MM440 pulmonary rehabilitation claims by billing medical specialty in Korea from 2017 to 2021. Proportions were calculated using MM440 claim counts classified by billing specialty. Rehabilitation Medicine, Internal Medicine, and Thoracic Surgery are presented separately, with the remaining specialties grouped as Other specialties. PR, pulmonary rehabilitation.

## Discussion

This nationwide study provides a population-level overview of pulmonary rehabilitation (PR) utilization and claim-based indicators of program delivery for chronic obstructive pulmonary disease (COPD) in Korea. Between 2017 and 2021, the proportion of patients receiving MM440-coded PR increased from 0.49% to 1.56%, whereas the mean number of MM440 claims per patient decreased over time. In 2021, most recipients (94.8%) had fewer than eight recorded MM440 claims. Because claims data do not capture program scheduling, actual treatment frequency, total duration, or completion, claim counts should be interpreted only as an indirect indicator of repeated reimbursed encounters rather than a direct measure of PR dose or participation in a complete program. Accordingly, fewer than eight claims should not be interpreted as evidence of non-adherence, discontinuation, or failure to complete PR.

In this study, “related clinical testing” was operationally defined as objective physiological or performance-related tests identifiable using reimbursed claim codes in the NHIS database. Although measures such as handgrip strength and respiratory muscle pressure testing are not COPD-specific, they function as reimbursable indicators of physical function or respiratory capacity within current Korean clinical practice (6, 7). The 6-minute walk test, a representative field measure of exercise capacity, could not be evaluated because its claim code (E7230) is also used for general oxygen saturation monitoring, making it indistinguishable in NHIS claims data.

Our findings extend previous Korean research based on the Health Insurance Review and Assessment Service (HIRA) database. Kim et al. reported increased PR utilization after insurance coverage was introduced, with lung cancer and COPD among the most common indications (4), and Choi et al. reported a COPD-specific PR utilization rate of 1.4% in 2019 (5). Using NHIS claims data, our study updates national trends through 2021 and adds patient-level socioeconomic and comorbidity information, alongside claims-based indicators that are directly relevant to implementation, including continuity and uptake of related clinical testing. The observed decrease in MM290 claims alongside increased MM440 claims may reflect a gradual shift toward more structured PR coding over time. Despite these increases, PR uptake in Korea remains low, consistent with international evidence that PR access and delivery are suboptimal even in high-income settings (12–14). These low utilization rates are likely multifactorial, reflecting system-level constraints and patient-level barriers, underscoring the need for implementation strategies that improve access, continuity, and quality measurement of PR (1, 15–20).

The matched comparison was intended to reduce basic demographic and geographic differences between MM440 recipients and non-recipients while descriptively examining real-world differences in healthcare utilization and clinical characteristics, rather than to estimate a causal effect of PR. Despite propensity score matching for age, sex, and region, meaningful imbalances remained between PR recipients and non-recipients in insurance category, hospital level, and healthcare utilization (SMD >0.1), indicating residual confounding and selection effects inherent to real-world PR delivery. PR recipients were more often enrolled in the Co-payment Reduction Program (48.7% vs. 35.1%; SMD 0.279) and were disproportionately treated at tertiary hospitals (81.2% vs. 50.0%; SMD 0.695), suggesting that access to structured PR may be concentrated in institutions with established rehabilitation infrastructure. In contrast, Medical Aid beneficiaries were more prevalent in the non-PR group (23.6% vs. 9.7%; SMD 0.380) despite minimal out-of-pocket expenses, implying that non-financial barriers may contribute to underuse. Higher admissions and longer respiratory-related hospital stays among PR recipients further suggest that PR was preferentially delivered to patients with greater clinical complexity or healthcare contact. Malignancy was the only comorbidity that differed between groups, reflected by higher malignancy-related claim counts in the PR group (11.9 vs. 6.2 claims per patient per year; SMD 0.327); however, the Co-payment Reduction Program covers multiple indications, and the NHIS dataset does not allow classification by specific eligibility criteria. In addition, inpatient versus outpatient PR and perioperative versus chronic disease–focused rehabilitation could not be separated, and these differences should therefore be interpreted primarily as reflecting real-world delivery patterns rather than causal determinants of PR use.

Regional variation in MM440-based PR implementation persisted over time. In 2017, higher rates were observed in Jeonbuk, Busan, and Ulsan, with Seoul also showing relatively high utilization. By 2021, Gangwon recorded the highest implementation rate, followed by Seoul, Busan, Jeonbuk, and Daejeon. Because NHIS data do not identify individual institutions, these regional patterns may partly reflect the activity of high-volume tertiary centers rather than uniform service availability within each region. The study period overlapped with the early COVID-19 pandemic in 2020–2021. MM440 utilization increased steadily during the pre-pandemic period, showed a relative plateau between 2019 and 2020, and increased again in 2021, whereas MM290 utilization declined after 2019. The pandemic may have affected healthcare access, hospital activity, and the delivery of face-to-face rehabilitation; however, the present claims data cannot distinguish pandemic-related effects from underlying temporal changes in PR implementation and coding practices. Further analyses incorporating post-pandemic years will be needed to determine whether these patterns represented temporary disruption or longer-term changes in PR delivery.

The distribution of MM440 claims by medical specialty showed that Rehabilitation Medicine accounted for the largest proportion, while the relative contributions of Internal Medicine and Thoracic Surgery were higher in 2021 than in 2017. Given the multidisciplinary nature of PR, coordination and referral pathways across specialties remain important to improve implementation and continuity of care (14).

Within claim-defined PR utilization, reimbursed objective clinical testing claims were recorded less frequently than MM440 claims. In 2021, the ratio of FX663 claims to MM440 claims was 11.2%, while the corresponding ratio for F6012 claims was 7.0%. Although the absolute numbers of these testing claims increased over time, they should be interpreted as annual claim-level measures rather than patient-level testing rates; the data cannot establish whether individual patients or discrete PR episodes lacked these assessments. Overall, these findings highlight the need for PR delivery models that extend beyond tertiary centers and incorporate standardized, reimbursable clinical testing to support quality measurement and continuity. Low referral and uptake are not unique to Korea; in an Australian audit conducted between 2020 and 2023, only 6.2% of PR referrals originated from primary care, mainly general practice (21). Strategies in Korea could therefore include strengthening referral pathways across care levels, incentivizing key objective assessments through reimbursement or quality indicators, and expanding home-based and tele-rehabilitation programs to improve access and support long-term participation. Although not COPD-specific, recent studies in cardiac and heart failure rehabilitation provide relevant translational evidence for technology-supported rehabilitation models (22–24). Home-based and hybrid telerehabilitation may reduce travel and time barriers, although engagement, therapeutic relationships, and implementation context remain important considerations (22). In addition, immersive virtual reality–based rehabilitation has been investigated as a strategy to improve rehabilitation adherence and patient engagement in heart failure and cardiac rehabilitation settings (23, 24). These approaches may inform future PR delivery models aimed at improving access and continuity, particularly for patients who face barriers to repeated center-based attendance.

This study provides a nationwide overview of PR utilization and related clinical testing practices among patients with COPD in Korea using NHIS claims data. The study period ended in 2021 because this was the most recent NHIS dataset available when the study was initiated. Limitations include the claims-based COPD definition without spirometric confirmation, symptom burden, medication information, or disease severity. To our knowledge, the exact algorithm used in this study has not been specifically validated against clinical or spirometric COPD diagnoses. Therefore, diagnostic misclassification is possible, and the sensitivity and specificity of cohort identification cannot be determined from the present data. Other limitations include the inability to capture the 6-minute walk test, absence of patient-reported outcomes such as CAT, claim-level rather than patient-level assessment of testing, and inability to distinguish inpatient from outpatient PR. Because discrete PR episodes could not be identified, the temporal relationship between PR and testing claims could not be determined. In addition, the data did not capture treatment frequency, total program duration, completion status, or participation in a structured PR program; therefore, the number of PR claims should be interpreted only as an indirect proxy for continuity rather than a direct measure of PR dose or completion. Accordingly, fewer than eight claims cannot be interpreted as non-adherence, discontinuation, or failure to complete PR. Although propensity score matching was performed for age, sex, and region, residual imbalances remained in hospital level, insurance status, healthcare utilization, and malignancy-related claims. Thus, residual confounding, selection bias, and confounding by indication cannot be excluded, and the matched comparison should not be interpreted causally.

In conclusion, claim-defined PR use among Korean patients with COPD increased after insurance coverage but remained low, and related clinical testing was applied infrequently. These findings identify persistent gaps in access and objective quality measurement within the current reimbursement-based delivery system and provide evidence to guide future PR implementation strategies in Korea.

## Data Availability

The datasets presented in this article are not readily available because the datasets analyzed in this study are owned by the National Health Insurance Service (NHIS) of Korea and are subject to strict data usage agreements. As the authors do not have the authority to redistribute the raw data, the datasets are not available by request to the corresponding author. However, access to the data can be requested through the National Health Insurance Sharing Service (https://nhiss.nhis.or.kr) upon approval of a research proposal and ethical review. Requests to access the datasets should be directed to https://nhiss.nhis.or.kr.

## Supplementary material

SUPPLEMENTARY FIGURE S1 Study population construction and annual identification of pulmonary rehabilitation-related claims among patients with COPD, 2017–2021. Eligible patients were adults aged ≥40 years with KCD-8 codes J43–J44, excluding J43.0, recorded as a primary or secondary diagnosis. Annual analytic populations and the numbers of patients with at least one MM440 or MM290 claim are shown. Stage-specific exclusion counts were not available in the final analysis output and were therefore not retrospectively reconstructed.
